# A Validated HPTLC-Densitometric Method for Assay of Aucubin in Vitexagnus-castusL.

**Published:** 2011

**Authors:** Homa Hajimehdipoor, Maryam Shekarchi, Morteza PiraliHamedani, Zahra Abedi, Homeyra Zahedi, Maral Shekarchi, Ahmad Reza Gohari

**Affiliations:** a *Traditional Medicine and MateriaMedica Research Center, ShahidBeheshti University of Medical Sciences, Tehran, Iran.*; b *Department of Traditional Pharmacy, School of Traditional Medicine, ShahidBeheshti University of Medical Sciences, Tehran, Iran.*; c *Food and Drug Laboratory Research Center and Food and Drug Control Laboratories, MOH and ME, Tehran, Iran.*; d *Department of Medicinal Chemistry, Faculty of Pharmacy, Tehran University of Medical Sciences, Tehran, Iran.*; e *Medicinal Plants Research Center, Faculty of Pharmacy, Tehran University of Medical Sciences, Tehran, Iran.*

**Keywords:** Aucubin; Chaste tree, HPTLC-densitometry, *Vitexagnus-castus*, Validation

## Abstract

*Vitexagnus-castus*L. is a medicinal plant which is used in several dosage forms for women hormonal disorders and standardized according to the iridoids or flavonoids content. Aucubin, an iridoid glycoside, considered as a marker in some formulations. In this research, a thin layer chromatographic method with densitometric detection has been developed for quantitative determination of aucubin in chaste tree fruits. Chromatographic separation was performed using silica gel high performance thin-layer chromatography (HPTLC) plates with ethyl acetate-methanol-water 77 : 15 : 8 as mobile phase. Chromatograms were visualized using *p*-dimethylaminobenzaldehyde as reagent. Aucubin R_F_-value was about 0.5 and spots were scanned at 580 nm through a mercury lamp. By using this method, the amount of aucubin was found 43.5 mg/100 g of dried plant fruits. The method was validated for selectivity, linearity (r^2^ = 0.997, 20-100 μg/mL), precision (intra-day < 4.9, inter-day < 7.2) and accuracy measured via determination of recovery (95-98%). The limit of detection and limit of quantization were found 6.6 and 20 μg/mL, respectively. This methodology was found to be precise with respect to the validation parameters. It is simple and convenient and could be applicable to the routine determination of aucubin in different *Vitexagnus-castus*L. samples.

## Introduction

*Vitexagnus-castus*L. (chaste tree) is a small shrub, native to Greece and Italy and has been naturalized to warm climates in the United-states. Its peppery fruit has been used as medicine for at least two thousand years. According to the historical reports, it has been used to treat hangovers, flatulence, fever and constipation. It was also recognized to bring on menstruation and relieve uterine cramps. Fruits are recommended not only as an emmenagogue but also as a lactation stimulator. Nowadays, chasteberry is used to condition the female reproductive system that may stem from latent hyperprolactinemia or corpus luteum insufficiency (1, 2). The commission E approved the use of chaste tree fruit for irregularities of the menstrual cycle, premenstrual complains and mastodynia (3). The plant fruits include the flavonoids (casticin, penduletin and chrysophanol D), alkaloids (viticin), iridoids (aucubin and agnuside) and volatile oil (1-4). Many commercial dosage forms have been made using chaste tree fruits. They are standardized according to their iridoid or flavonoid contents (5, 6). Aucubin is an iridoid glycoside which can be selected as a marker component in some drug formulations. Different methods can be employed to quantitative analysis of plants’ components specially Gas Chromatography (GC) and High-performance liquid chromatography (HPLC) which are most popular. These two techniques need the plant matrixes to be cleaned up which is usually a prolonged and difficult process and using special instruments and materials are obligated (7-9). Moreover, GC is a technique that is only used for assay of volatile compounds directly. In order to analyze the non-volatile constituents, derivatization is necessary which takes time and is not cost-benefit. In addition, recovery percentage should be considered (10). Therefore, developing a new simple, rapid and accurate method for quantization of plant components is important. High performance thin-layer chromatography (HPTLC) is one of the useful methods which doesn’t need tedious clean up and by using appropriate mobile phases and reagents, all interfering agents will be omitted. It is a very rapid, accurate and precise chromatographic technique for many herbal components assay and can be used for routine quality control of herbal products (11-15). Thin layer chromatography (TLC) is a popular method especially for screening and identification of many substances. This technique enables parallel separation and direct comparison of standards with sample components by using various chromatographic systems, as well as development and detection modes. By using TLC technique, determination of semi-volatile substances could be easily carried out as well (16). In this research, quantitative analysis of aucubin in chaste tree fruits by means of HPTLC-densitometry method has been developed and validated for selectivity, linearity, accuracy, intra and inter-day precision.

## Experimental

*Plant material*

Fruits of *Vitexagnus-castus*were collected in July 2008 From Sabzevar (Khorasan province) and identified by M. Khatamsaz, Research Institute of Forests and Rangelands, Tehran. 

*Chemicals*

Aucubin reference standard was purchased from ROTH (Germany). All the solvents, *p*-dimethylaminobenzaldehyde and HPTLC silica gel 60 F_254_ plates (20 × 20 cm) were obtained from Merck. (Merck, Germany).

*Preparation of sample solutions*

Samples (1 g) of dried and milled plant fruits were extracted with methanol (3 × 3 mL) by being shaken at room temperature for ten min and centrifuged. The combined supernatants were diluted to 10 mL with the same solvent (5).

*Preparation of standard solutions*

Aucubin reference standard (10 mg) was weighed accurately and diluted with methanol to 10 mL (stock solution, 1 mg/mL). Different dilutions (10-120 μg/mL) were prepared using stock solution. 

*Chromatographic procedure*

Densitometric evaluation of the spots was carried out using a Camag TLC scanner III in absorbance mode under wavelength of 580 nm (mercury vapor lamp). The scanner was combined with Wincats software (Camag, Switzerland) for the evaluation of densitometry results. HPTLC plates were pre-washed with methanol, dried for 1 h at 100°C and cooled. Samples and standard solutions (50 μL) were applied as 10 mm bands, 10 mm apart, 10 mm from the both sides and 20 mm from the bottom using CamagLinomat IV automatic sample applicator. Ethyl acetate : methanol : water (77 : 15 : 8) was used as mobile phase. The plates were developed in a saturated vertical-developing chamber at room temperature for 1 h. The development distance was 12 cm and the development time was about 2 h. After the development, the plates were dried and dipped in a reagent solution (*p*-dimethylaminobenzaldehyde 10 mg/mL in HCl 1 N). In order to detect the spots, plates were heated on a digital hot plate, at 120°C for 10 min. 

**Figure 1:**
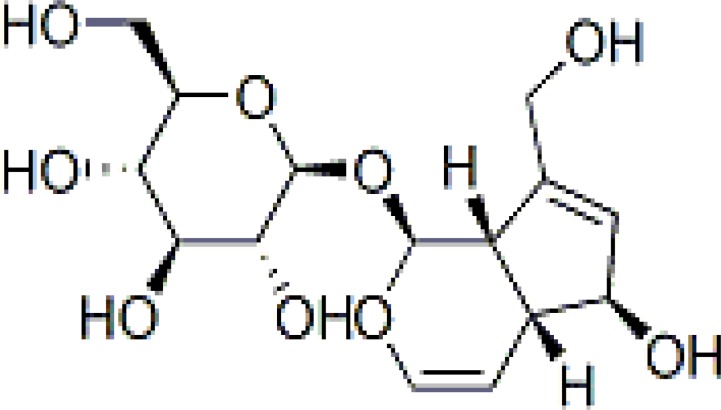
Structure of aucubin

*Calibration curve for aucubin*

Fifty μL of each standard solution was applied on the HPTLC plates (triplicate). The plates were dried, developed and analyzed as described. The standard calibration curve was generated using the regression analysis with Microsoft Excel.

*Validation procedure*

The method was validated in terms of linearity, selectivity, intra- and inter-day precision and accuracy.

*Linearity*

The linearity of the method was evaluated by analysis of seven aucubin standard solutions with concentrations of 10, 20, 40, 60, 80, 100, and 120 μg/mL. The solutions were applied on the same plate. The plate was developed using the above-mentioned mobile phase.

*Limit of detection and Limit of quantization*

Limit of detection (LOD) and Limit of quantization (LOQ) were calculated using 3.3σ/s and 10σ/s expressions respectively, in which “σ” is standard deviation of blank response the intercept standard deviation and “s” is the slope of calibration curve.

*Selectivity*

In order to confirm the selectivity of the proposed method for the analysis of aucubin, 10 μL of sample solution was applied on the plate and two dimensional TLC were performed using the same mobile phase.

*Precision*

Instrument precision was checked through scanning the aucubin band (50 μg/mL) for ten times and CV% was calculated. Intra-day precision was determined through analyzing the sample solutions of 0.8, 1 and 1.2 g of fruit in 10 mL solvent (three extraction for each concentration and three spots for each extract) on the same day, while inter-day precision was determined through analyzing the corresponding solutions on three consecutive days. The repeatability of sample application was assessed by spotting 50 μL of standard solution (50 μg/mL) for three times on a plate followed by visualization and recording the peak area for three spots.

*Accuracy*

The accuracy of the method was tested by determination of recovery at two levels (in triplicates) after 100 and 300 μg addition of original aucubin into the sample matrix before extraction and the amount of aucubin was determined.

## Results and Discussion

*Chromatographic conditions*

**Figure 2:**
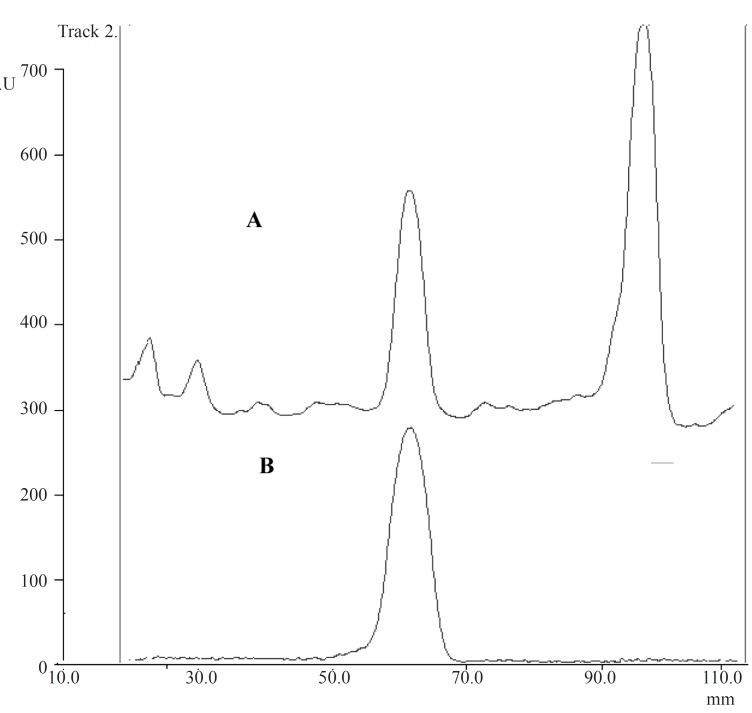
(A) TLC-densitometric chromatograms of Chaste tree extract (B) aucubin standard solution.

For spotting the chaste tree extract, different volumes (10-100 μL) were tested and volume of 50 μL of samples was chosen for the experiment. Between several investigated mobile phases, ethyl acetate : methanol : water (77 : 15 : 8) in a saturated chamber was found to be the most selective and repeatable for the determination of aucubin in *Vitexagnus-castus *extract. Among reagents tested for visualization of spots, *p*-dimethylaminobenzaldehyde was the reagent of choice. Under this condition, only two blue spots were observed: aucubin (R_F_ 0.5) and agnuside (R_F_ 0.8). These two components were well separated in the chromatogram and could be easily scanned. The identity of aucubin peak was confirmed by comparing the R_F_ of the peak from the standard with those of the corresponding peak in the sample as shown in Figure 2. For the visualization of the spot, two methods were used: spray the reagent on the plates and dipping the plate in the reagent tank. The second method was found to be more repeatable. In addition, for heating the plates, different instruments including oven, electronic hot air blower and digital heater plate were tried the last of which showed to be more reproducible. By using this method, the amount of aucubin was found to be 43.5 mg/100 g dried powder of *Vitexagnus-castus *fruits.

*Validation*

Validation data for the presented method are summarized in Tables 1 and 2. The method was specific since after the two-dimensional chromatography, the same two spots (aucubin and agnuside) were present and no interfering spot was observed. The results showed linear relationship between the peak areas and the aucubin concentrations. The plot was linear in the range of 20-100 μg/mL (y = 0.2075 x + 3.45, r^2^ = 0.997). A graph of residuals against the concentrations of aucubin was also plotted. It was observed that the residuals were distributed both above and blow the zero residuals line. LOD and LOQ were found to be 6.6 and 20 μg/mL, respectively. For intra- and inter-day precision, variation coefficients were 3.0-4.9% and 5.0-7.2%, respectively. These results can be compared with a recent work (13) on the HPTLC of acteoside in *Plantago palmate *(UV detection at 334 nm; mobile phase, ethyl acetate : water : formic acid (90 : 5 : 5)). The researchers measured CV% at one level of concentration, from six spots of standard solution repeated on three plates (one plate per day; CV% of the daily averages: 2%). Another study on eugenol in *Cinnamomum tamala *showed 0.98% and 1.32% for intra- and inter-day precision (17), respectively. These values are significantly less than the CV% measured in the present work.

**Table 1:** Method validation data for quantization of aucubin in chaste tree by HPTLC-densitometric method

**Parameters**	**Results**
Spotting repeatability (n = 3, CV%)	2.1
Peak area measurement repeatability (n = 10, CV%)	0.6
LOD (μg/mL)	6.6
LOQ (μg/mL)	20
Selectivity	selective
Linearity (r^2^)	0.997
Range (μg/mL)	20-100
Intra-day precision 80% (n = 3, CV%)	4.9
Intra-day precision 100% (n = 3, CV%)	3.0
Intra-day precision 120% (n = 3, CV%)	3.5
Inter-day precision 80% (n = 3, CV%)	5.5
Inter-day precision 100% (n = 3, CV%)	5.0
Inter-day precision 120% (n = 3, CV%)	7.2

**Table 2:** Recovery study of aucubin in chaste tree by HPTLC-densitometric method

Amount of aucubin in sample (μg/mL)*	**Added (μg/mL)**	Found (μg/mL)*	**Recovery (%)**
41.7 ± 0.71	10	49.4 ± 0.41	95.5
	30	70.5 ± 0.53	98.3

*: Mean ± SD (n = 3)

This difference is due to performing the method validation procedure on plant material (this work) and standard solution (other works). It is obvious that method validation on standard material could not represent the variability which is expected from applying the analytical method to the real sample.

## Conclusion

TLC and TLC-densitometry have been largely investigated for purity testing, pharmaceutical dosage form assay and herbal fingerprinting (18). These methods are flexible and cost-effective and present the advantage of the simultaneous processing of standards and samples with versatile detection possibilities, including a great variety of post-chromatographic derivatization reagents due to the short analysis time and low solvent consumption. The proposed method has been shown to be highly suitable for the analysis of aucubin in *Vitexagnus-castus*L. with respect to the selectivity, linearity, accuracy, repeatability and speed. 
